# Autochthonous and imported giardiasis cases: An analysis of two decades of national surveillance data, Germany, 2002 to 2021

**DOI:** 10.2807/1560-7917.ES.2024.29.20.2300509

**Published:** 2024-05-16

**Authors:** Franziska Hommes, Achim Dörre, Susanne C Behnke, Klaus Stark, Mirko Faber

**Affiliations:** 1 Department of Infectious Disease Epidemiology, Robert Koch Institute, Berlin, Germany; 2 Postgraduate Training for Applied Epidemiology (PAE), Robert Koch Institute, Berlin, Germany; 3 ECDC Fellowship Programme, Field Epidemiology path (EPIET), European Centre for Disease Prevention and Control (ECDC), Stockholm, Sweden

**Keywords:** Giardiasis, epidemiology, surveillance, imported disease

## Abstract

**Background:**

*Giardia duodenalis* is a major cause of gastroenteritis globally, and is the most common food- and waterborne parasitic infection in Europe.

**Aim:**

To describe the epidemiology of reported acute giardiasis cases in Germany and compare demographic and clinical characteristics between imported and autochthonous cases.

**Methods:**

We conducted a descriptive analysis of giardiasis cases that fulfilled the national case definition and were reported between January 2002 and December 2021. We defined an imported case as having at least one place of exposure abroad in the 3–25 days before symptom onset. We analysed case numbers and incidence by age, sex, month reported and geographic region, both overall and stratified by autochthonous and imported cases.

**Results:**

From 2002 to 2021, 72,318 giardiasis cases were reported in Germany, corresponding to a mean annual incidence of 4.4 per 100,000 population. Annual incidence gradually decreased since 2013, declining sharply during the COVID-19 pandemic in 2020–21. Of 69,345 cases reported between 2002 and 2019, 35% were imported. Incidence of autochthonous cases (overall yearly mean: 3.1/100,000) was highest in males and young children (< 5 years); imported cases were predominantly adults aged 20–39 years. We identified seasonal patterns for imported and autochthonous cases.

**Conclusions:**

Giardiasis in Germany is typically assumed to be imported. Our data, however, underline the importance of autochthonous giardiasis. Travel advice might reduce imported infections, but prevention strategies for autochthonous infections are less clear. Dietary, behavioural and environmental risk factors need to be further investigated to enhance infection prevention measures for autochthonous giardiasis.

**Key public health message**

**What did you want to address in this study and why?**

Giardiasis is the most common food- and waterborne parasitic disease in Europe, causing abdominal cramps and acute/chronic diarrhoea. In Germany, giardiasis is typically assumed to be associated with travel abroad, however there is a notable occurrence in people without travel history. We wanted to identify the main risk groups and long-term trends, and understand how to guide prevention strategies.

**What have we learnt from this study?**

Giardiasis incidence has decreased considerably during the past 10 years in Germany, particularly in children. About one third of all cases were imported. Young children, 20–55-year-olds and males were most affected by non-travel-associated infections. We also observed geographical and seasonal patterns, indicating that food-borne, environmental and person-to-person transmission contribute to non-travel-associated infections in Germany.

**What are the implications of your findings for public health?**

Our findings should help to raise awareness among clinicians and public health experts about the incidence of non-travel-associated giardiasis, and particularly affected population groups. Given the current epidemiology, both classical epidemiological and molecular typing studies are necessary to clarify infection sources, define risks and inform prevention strategies.

## Introduction

*Giardia duodenalis* (syn. *G. lamblia, G. intestinalis*) is a major cause of gastroenteritis worldwide. In Europe, giardiasis is the most commonly reported food- and waterborne parasitic disease and was ranked among the top 10 most important food-borne parasites for public health [1,2]. The most common transmission routes, which also cause outbreaks of giardiasis in Europe, include waterborne, food-borne and person-to-person transmission, exposure during travel and direct faecal exposure such as through contact with children of diaper-wearing age [3].

Over 80% of current European Union/European Economic Area (EU/EEA) countries have implemented a surveillance system for giardiasis [4]. According to the 2019 Annual Epidemiological Report from the European Centre for Disease Prevention and Control (ECDC), 18,004 confirmed cases of giardiasis were reported in the EU/EEA, resulting in an overall incidence of 5.2 cases per 100,000 population. Notably, the highest incidence was observed in children 0–4 years old [2]. In Germany, a national surveillance system has captured information about acute cases since 2001. Between 2015 and 2019, Germany reported the second highest number of cases in the EU/EEA, averaging around 3,500 cases annually. In 2019, the age-standardised rate in Germany was 4.1 cases per 100,000 population, slightly below the overall age-standardised rate of 5.3 in the EU/EEA [2].

In Europe, giardiasis is generally considered to be mainly an imported disease [2,5]. However, 69% of reported giardiasis cases in EU/EEA were autochthonous cases, with respective proportions strongly varying between countries [2]. An intensified surveillance and case–control study conducted in Germany in 2007 and 2008 showed that nearly half of the cases had acquired an infection in Germany. The study identified that male sex, immunosuppression and eating green salad daily were associated with symptomatic *G. duodenalis* infection [6].

An in-depth analysis describing both the recent epidemiology of giardiasis in Germany and the epidemiology over a long time period is lacking. Here, we aimed to describe the numbers of symptomatic giardiasis cases in Germany reported in the national surveillance system in 2002–21 by time, place and person, and to compare the demographic and clinical characteristics between imported and autochthonous cases. In addition, based on a thorough description of the population groups most affected by giardiasis in Germany, we aimed to generate hypotheses about risk factors for autochthonous *Giardia* infections.

## Methods

### Data collection

Giardiasis is a notifiable disease in Germany, according to the national infection protection act of 2001 [7]. Direct or indirect evidence of *G. duodenalis* infection in patients is reported to the local public health department by the identifying laboratory. The local public health departments complete information about the case by requesting additional information from the diagnosing laboratories, patients or treating physicians including demographic characteristics, date of symptom onset, travel history and hospitalisation. Case information is then verified according to the German national case definition (see Box), anonymised, and electronically transmitted from local health departments via state health departments to the Robert Koch Institute (RKI), Germany’s national public health institute. For the purpose of this study, we defined an imported case as an individual with symptoms that fulfils the case definition and with at least one reported place of exposure abroad in the 3–25 days (the common range of the incubation period) before symptom onset. We classified all other cases as autochthonous, i.e. cases for which Germany was reported as the only place of exposure, or without information on the place of exposure.

Box **Germany’s national case definition for acute Giardiasis, Robert Koch Institute, 2019 [33]**

A confirmed case must meet the clinical criteria and be confirmed either through laboratory testing or epidemiological investigation as follows:

**Clinical picture** of acute giardiasis, defined as presence of at least one of the following symptoms:

• Diarrhoea

• Abdominal pain

• Flatulence

OR

• Death because of giardiasis

**Laboratory confirmation**, defined as a positive outcome of at least one of the following:

• Antigen detection

• Microscopy

• Detection of *G. duodenalis* DNA using nucleic acid tests (since 2015)

**Epidemiological confirmation**, defined as detection of at least one of the following, considering the incubation period (3–25 days, occasionally longer)

• Epidemiological link with a laboratory-confirmed giardiasis case through human-to-human transmission

OR

• A common source of exposure (bathing in a laboratory-confirmed contaminated body of water, or consuming laboratory-confirmed contaminated food or water)

### Data analysis

We conducted a descriptive analysis of cases that fulfil the national case definition reported through the national surveillance system in Germany from 1 January 2002 to 31 December 2021. We analysed case numbers and incidence, both overall and stratified for autochthonous and imported cases. We further stratified the data by year of notification, age, sex, region of residence, clinical symptoms, hospitalisation status, likely country of exposure (for imported cases) and laboratory methods. We conducted a sub-analysis for the years 2020–21 to describe patterns during the sharp decline of case numbers during the COVID-19 pandemic.

We used R version 4.1.3, Rstudio version 2022.07.2 + 576 and MS Excel for data analysis. We created the map with R version 4.1.3 and the package tmap [8]. The map is based on SHP-Files by GeoBasis-DE / BKG (2022).

## Results

**Figure 1:**
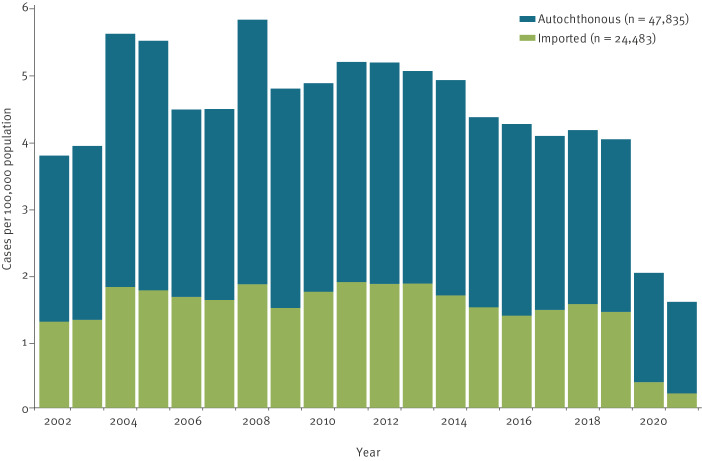
The incidence of reported autochthonous vs imported giardiasis cases by year of notification, Germany, 2002–2021 (n = 72,318)

Between 2002 and 2021, a total of 72,318 giardiasis cases (56.1% male; median age: 37 years) were reported in Germany, corresponding to a mean annual incidence of 4.4 per 100,000 population (2002–19: 4.7/100,000; 2020–21: 1.8/100,000). The annual giardiasis incidence showed a gradual increase between 2002 and 2012 with peaks in 2004, 2005 and 2008, followed by a decrease between 2013 and 2019 and a sharp decline during the COVID-19 pandemic in 2020 and 2021 (Figure 1). Of all cases reported between 2002 and 2019, 23,986 of 69,345 (34.6%) were imported. The proportion of imported cases before the COVID-19 pandemic ranged from 32.0% (1,447/4,519) in 2005 to 37.4% (1,278/3,418) in 2018, and decreased to 19.1% (319/1,669) and 13.7% (178/1,304) in 2020 and 2021, respectively.

### Laboratory methods

**Figure 2:**
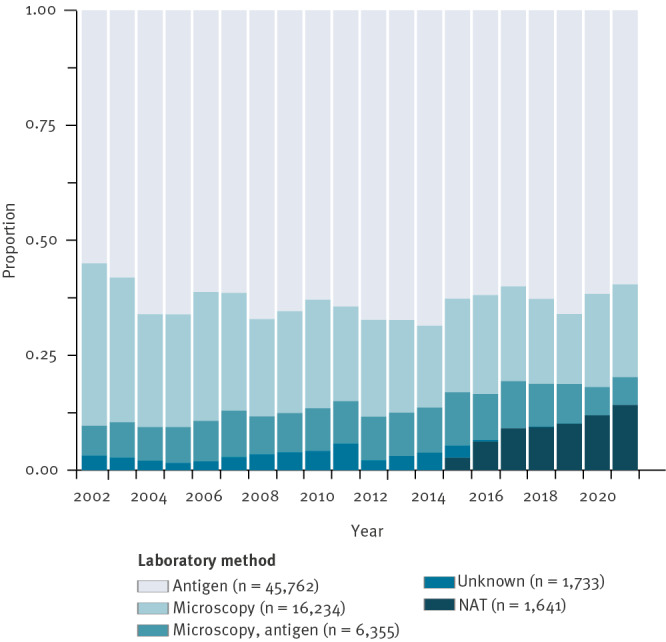
Reported giardiasis cases by year of notification and laboratory method, Germany, 2002–2021 (n = 71,863)

Most cases notified in 2002–21 were laboratory-confirmed (71,863/72,318; 99.4%). Among those, most were diagnosed using either antigen ELISAs (45,762/71,863; 63.7%) or microscopy (16,234/71,863; 22.6%) (Figure 2). The proportion of nucleic acid tests increased steadily since this method was included in the national case definition, from 2.7% (97/3,582) in 2015 to 14.0% (182/1,302) in 2021, while diagnosis using only microscopy has declined from 35.3% (1,086/3,078) in 2002 to 19.9% (259/1,302) in 2021.

NAT: nucleic acid test.

Rarely reported combinations of diagnostic methods (< 0.2% of total number of reported methods) not shown: microscopy, NAT (n = 85); antigen, NAT (n = 42); microscopy, antigen, NAT (n = 11).

### Epidemiology of autochthonous giardiasis cases

In 2002–19, 65.4% (45,359/69,345) of all cases were autochthonous cases, corresponding to a mean annual incidence of 3.1 cases per 100,000 population. The mean annual incidence of autochthonous cases increased from 2002 to 2008, with peaks in 2004, 2005 and 2008, plateaued between 2009 and 2012 and decreased since 2013 (Figure 1).

**Figure 3:**
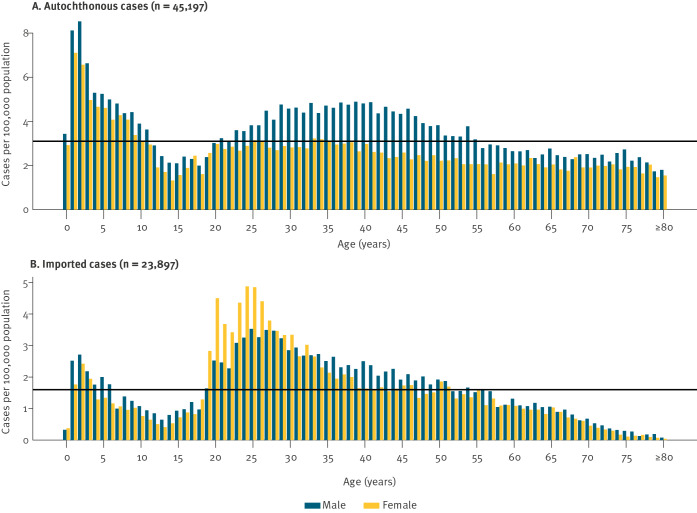
Incidence of giardiasis cases by age and sex, Germany, 2002–2019 (n = 69,094)

Compared with the mean annual incidence in all autochthonous cases reported in 2002–19 (3.1/100,000), children under the age of 5 years had the highest incidence of up to 8.5 cases per 100,000 population (Figure 3). A higher level compared to the mean of all age groups was also evident in 20–55-year-olds, with an incidence of up to 4.9 cases per 100,000 population. The difference in incidence between sexes was most evident in 27–57-year-old adults (male-to-female ratio of up to 2.0). While the incidence in males rose steadily from 15 years until the age of 35–40 years (up to 4.9 cases/100,000 population) and slowly declines thereafter, females had the highest incidence around the age of 20–39 years (up to 3.2 cases per 100,000 population), slowly declining with age thereafter. Age-specific incidence in adolescents between 12 and 19 years and adults above 60 years was comparatively low.

Black horizontal lines show the respective mean incidence of giardiasis cases from 2002–19.

#### Incidence by age and year of notification

**Figure 4:**
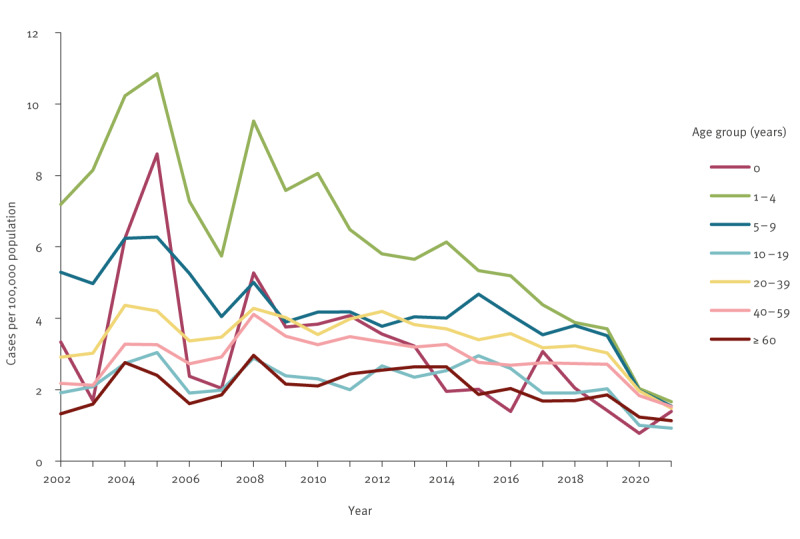
The incidence of autochthonous giardiasis cases by age group and year of notification, Germany, 2002–2021 (n = 47,810)

While the overall incidence of giardiasis declined over the last 2 decades, the stratified analysis of autochthonous cases by age group showed the most substantial decrease in the incidence in children below the age of 5, which decreased by more than 70% from 5.73 per 100,000 in 2002 to 1.62 per 100,000 in 2019 (Figure 4). The giardiasis incidence in 5–9-year-olds also showed a decrease over time with a 1.5-fold reduction from 2002 to 2019. The incidence in older age groups slightly decreased. Since 2015, the incidences of all age groups have nearly converged. The incidence proportion of both sexes in various age groups (indicated in Figure 3) did not show specific trends over time.

#### Hospitalisation, deaths and clinical symptoms

Of 45,359 autochthonous giardiasis cases notified in 2002–19, 5,218 cases (11.5%) were hospitalised. The hospitalisation proportion ranged between 8.5% (196/2,303) in 2006 and 14.1% (298/2,118) in 2019. Hospitalisation was highest for older people (438/1,280; 34.2% in persons > 79 years old and 1,259/6,515; 19.3% in 60–79-year-olds) and children (340/1,754; 19.4% in 10–14-year-olds and 234/1,355; 17.3% in 0–1-year-old infants).

Three deaths because of an acute giardiasis infection were reported in 2002–19 (3/42,733 cases with reported hospitalisation status; 0.01%).

In 2002–19, autochthonous cases reported diarrhoea (36,707/45,359; 80.9%), pain (23,770/45,359; 52.4%), flatulence (11,074/45,359; 24.4%) and other symptoms (not specified) (3,074/45,359; 6.8%). Stratification by age and sex did not reveal relevant differences in symptoms.

For autochthonous cases, the median duration between the onset of symptoms and being confirmed as a case was 12 days.

#### Geographical distribution

**Figure 5:**
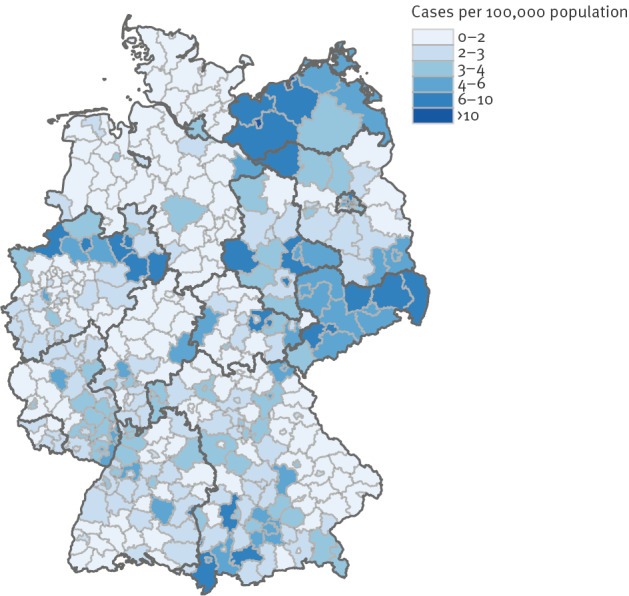
The incidence of autochthonous giardiasis cases at the district level, Germany, 2002–2019 (n = 45,345)

The geographical distribution of the incidence of autochthonous cases reported from 2002 to 2019 on the district and state level showed a heterogenous pattern (Figure 5). Besides smaller regional clusters, there were regions with comparatively high incidence, especially in eastern Germany and some parts of western Germany. The mean annual incidence in 2002–19 varied between 0.1 and 13 cases per 100,000 population in the different districts in Germany.

The borders of the 411 districts are shown in light grey, and the darker outlines show the borders of the 16 states in Germany.

#### Seasonality

**Figure 6:**
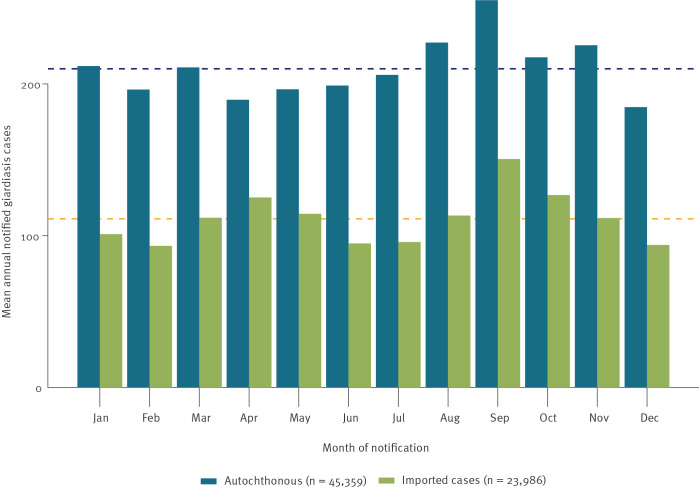
The mean number of reported autochthonous vs imported giardiasis cases by month of notification, 2002–2019 (n = 69,345)

Monthly autochthonous giardiasis cases reported between 2002 and 2019 showed two smaller peaks in January and March and a prolonged peak in August–November, exceeding the mean of autochthonous cases reported in 2002–2019 (Figure 6).

The upper dashed line (autochthonous cases) and lower dashed line (imported cases) show the mean number of giardiasis cases, 2002–19.

### Epidemiology of imported giardiasis cases

In 2002–19, 34.6% (23,986/69,345) of all giardiasis cases were imported, corresponding to a mean annual incidence of 1.6 cases per 100,000 population (Figure 1). Before the COVID-19 pandemic, the proportion of imported cases varied between 31.3% (n = 1,242) of all cases in 2009 and 37.4% (n = 1,278) in 2018.

The age distribution of imported giardiasis cases showed two peaks regarding incidence: a smaller peak in 1–5-year-old children (up to 2.7 cases/100,000 population) and younger adults aged 20–39 years old (up to 4.9 cases/100,000 population) (Figure 3). The incidence in males was higher than in females in most age groups, except for adults aged 19–32 years old.

#### Hospitalisation, deaths and clinical symptoms

Of 23,986 imported giardiasis cases notified in 2002–19, 1,384 cases (5.8%) were hospitalised. The hospitalisation proportion ranged from 4.5% (64/1,429) in 2010 to 8.1% (101/1,251) in 2015. Hospitalisation was highest for older imported cases (13/57, 22.8% in persons ≥ 79 years old) and children (47/370, 12.7% in 11–14-year-olds and 35/325, 10.7% in 0–1-year-olds). No deaths from giardiasis were reported in imported cases in 2002–19.

In 2002–19, 21,101 of 23,986 imported cases (87.9%) reported diarrhoea, pain (13,941/23,986; 58.1%), flatulence (7,683/23,986; 32.0%) and other symptoms (2,322/23,986; 9.7%). The median duration between the onset of symptoms and being reported as a case was 24 days.

#### Seasonality

Imported giardiasis cases showed a clear seasonality with peaks in April (March–May) and in September (August–October), with the peaks exceeding the mean of imported cases reported in 2002–19 (Figure 6).

#### Exposure countries

Countries most frequently reported as places of exposure in 2015–19 by imported cases were located in Europe, Asia, Africa and South America. The five countries most often reported as places of exposure abroad in 2015–19 were India, Spain, Thailand, Colombia and Türkiye (Table).

**Table:** Top 15 countries reported as place of exposure in imported giardiasis cases, Germany, 2015–2019 (n = 6,049 cases)

Exposure country	Imported giardiasis cases	
	n	%
India	1,231	20.4
Spain	258	4.3
Thailand	241	4.0
Colombia	193	3.2
Türkiye	192	3.2
Italy	178	2.9
Egypt	150	2.5
Tanzania	144	2.4
Mexico	129	2.1
Peru	122	2.0
Nepal	121	2.0
Indonesia	118	2.0
Morocco	105	1.7
Cuba	100	1.7
Brazil	94	1.6

Multiple mentions per case possible.

### Effects of the COVID-19 pandemic

During the COVID-19 pandemic in 2020 and 2021, the mean annual giardiasis incidence decreased to 1.8 cases per 100,000 population, from 4.1 cases per 100,000 population in the previous 5-year period (Figure 1). The proportion of imported cases declined from 35% in the previous 5-year period to 19% in 2020 and 14% in 2021.

In 2020–21, the mean annual incidence was highest in children aged < 5 years old (up to 1.1 autochthonous and 0.4 imported cases/100,000 population) and adults aged 20–39-years old (up to 0.9 autochthonous cases and 1.1 imported cases/100,000 population). Autochthonous cases had a higher male-to-female ratio (1.5) than imported cases (1.2).

The hospitalisation proportion of autochthonous cases was higher in 2020 (189/1,350; 14.0%) and 2021 (179/1,126; 15.9%) than in the previous 5-year time period (1,465/11,103; 13.2%). The hospitalisation proportion of imported cases was lower in 2021 (7/178; 3.9%), but higher in 2020 (21/319; 6.6%) than in the previous 5-year time period (395/6,049; 6.5%). No deaths from giardiasis were reported in 2020 and 2021.

## Discussion

Our study provides a thorough description of giardiasis cases reported in Germany covering a long time period of 20 years. We found that infections with *G. duodenalis* are a common cause of both imported and autochthonous gastroenteritis, and are the fifth most common cause of gastroenteritis in Germany among notifiable pathogens (after *Norovirus*, *Campylobacter*, *Rotavirus* and *Salmonella,* and before *Yersinia enterocolitica*, *Clostridioides difficile*, enterohaemorrhagic *Escherichia coli* and *Cryptosporidium parvum*) [9].

Our results provide additional insights into the broader epidemiology of giardiasis in Europe. While the notification rate in EU/EEA remained rather stable between 2013 and 2019 [2,10], we observed a decreasing trend of giardiasis cases in Germany since 2013. A decreased giardiasis incidence across all age groups was also described for the time period 1995–2016 in the United States [11].

Imported cases especially affected younger adults 20 to 39 years and predominantly females in this age group. The incidence of autochthonous cases is still highest in young children, despite its sharp decline over the last 2 decades. The decline might have both mirrored as well as driven the overall incidence decline of autochthonous giardiasis cases in Germany since 2013. Spending time at kindergarten is likely a main avenue of exposure to *G. duodenalis* infection for children under 5 years of age [12]. Potential factors contributing to the observed decline in children under 5 years might be a gradual improvement of infection prevention and control measures since the implementation of the infection protection act in Germany, which also applies to kindergartens, schools and other childcare centres, as well as intensified hygiene promotion initiatives such as training and ‘Hygiene Tips for Kids’ [13,14]. Alternative hypotheses, such as reduced *Giardia* exposure because of a change in dietary habits or an actual decreased prevalence of the parasite in food items preferred in this age group should be considered as well. Of note, other gastrointestinal pathogens, such as *Campylobacter*, *Yersinia*, *Shigella* and *Salmonella*, exhibited a similar pattern [15].

Despite an overall longstanding trend of a rising proportion of children under 3 years of age visiting daycare centres, the proportion in eastern federal states of Germany is still higher than in other parts of Germany [16]. This may have contributed to the observed geographical pattern of autochthonous giardiasis.

In previous studies, both nationally [6] and internationally [2,17,18], cases were more frequently male than female. This is in line with our results on autochthonous cases, in particular in the age groups 20–59 years. The higher incidence in autochthonous adult males may partially be explained by sexual transmission routes among men who have sex with men [19,20]. Our data showed that adults 20–39 years old had the highest incidence of imported giardiasis, likely attributable to a higher proportion of travel to highly endemic destinations and travel styles with a higher risk of exposure [21]. Contrary to most age groups in both imported and autochthonous cases, women were overrepresented among imported cases aged 19–32 years. The reason is unclear but could be related to sex differences in vacation behaviour such as choice of destination, activities or food [22]. Other potential biological and behavioural factors that might have contributed to the observed male-to-female ratio in small children need to be further investigated.

We observed seasonal patterns both for autochthonous and imported cases. An overall rise of giardiasis cases in August–October with a peak in September has also been described for Europe [2,10]. The peak of imported cases in April and September corresponds to holiday seasons in Germany with travellers returning from countries with higher *G. duodenalis* endemicity.

The observed seasonal patterns for autochthonous cases correspond to evidence from the area of the Lower Rhine in West Germany, where the highest numbers of *Giardia* cysts were found in wastewater from September to January [23]. The peak of autochthonous cases in late summer and autumn might be partly due to secondary transmission from infected returning travellers. Increased recreational water activities likely contributes to the peak of autochthonous cases at the end of the summer holiday period, as it was observed in other countries [17,23]. Higher temperatures during the summer months and higher rainfall might also impact the peak of *Giardia* cysts in the environment during late summer [23,24]. Because of climate change-induced higher temperatures and extreme weather such as heavy rainfall and floods, the prevalence of *Giardia* cysts in water bodies and the risk of contamination of plant foods may increase further in the coming years [25,26].

Without adequate molecular typing tools available, which are a prerequisite for molecular source attribution studies, it is difficult to estimate the proportion of cases caused through secondary human-to-human, food-borne, environmental and other transmission routes [27]. Available typing methods, so far, lack discriminatory power and are not applied in routine diagnostics or molecular surveillance. Recent studies have, however, shown promise in using multilocus sequence typing (MLST) to successfully type certain subtypes, specifically assemblage A, of *Giardia lamblia* [28,29].

The distribution of foreign countries most frequently mentioned as places of exposure in imported giardiasis cases reflects the situation observed in travel clinics. It does not, however, represent the actual risk of acquiring an infection in those countries. An appropriate denominator, i.e. the number of people who travelled, is needed to estimate the risk of infection in each country; we investigated that factor in a separate study [30].

As with many other notifiable infectious diseases in Germany, we observed a strong decline of giardiasis incidence during the COVID-19 pandemic in 2020 and 2021 [31]. The notification rate for giardiasis in Europe decreased by 50% from 5.1 cases per 100,000 population to 2.5 in 2021 overall [32]. Factors that are likely to have reduced *G. duodenalis* infections and infection transmission include reduced healthcare-seeking behaviour of patients and potential underreporting of cases, national and international travel restrictions, and non-pharmaceutical measures such as increased hand hygiene, contact restrictions, interim closures of kindergartens, schools, swimming pools and other public places [31]. The strong incidence reduction suggests that the non-pharmaceutical measures against the spread of SARS-CoV-2 were also effective against other infections with person-to-person transmission, including parasitic diseases.

We acknowledge the common limitations associated with data collected through passive infectious disease surveillance systems, including under-reporting, incomplete data reporting and potential misclassification of cases. Nevertheless, given the large size of our dataset (> 72,000 observations), and the presence of a long-established and standardised reporting system, along with several opportunities for cross-verification of findings, the impact on the overall results of our analysis is likely low and important trends should be adequately represented. Furthermore, the surveillance system in Germany only captures acute infections of *G. duodenalis*. However, chronic infections should be considered as part of the overall health burden of giardiasis.

## Conclusion

The overall incidence of giardiasis has slightly declined over the last 10 years and age groups seem to have aligned in terms of infection risk. While imported infections can best be addressed through pre-travel-advice, the case for further preventing autochthonous cases is less clear: the observed seasonality, the variability in geographical distribution, and the distinct incidence difference between sexes and age groups, i.e. comparatively high incidence of autochthonous giardiasis in males and small children, may indicate the concurrent existence of different risk factors in different population groups. These include dietary, demographic, environmental and behavioural risks, which need more detailed investigation in future studies, e.g. using case–control designs. Despite a declining trend in incidence in recent years, the evolving epidemiology of giardiasis in Germany needs to be further monitored and investigated. Factors such as altered environmental conditions due to climate change, and changes in the application of laboratory methods for diagnosis and molecular typing, may affect the trends in reported giardiasis cases in the future.

## References

[1] Bouwknegt M Devleesschauwer B Graham H Robertson LJ van der Giessen JW Euro-FBP workshop participants . Prioritisation of food-borne parasites in Europe, 2016. Euro Surveill. 2018;23(9):17-00161. 10.2807/1560-7917.ES.2018.23.9.17-00161 29510783 PMC5840924

[2] European Centre for Disease Prevention and Control (ECDC). Giardiasis (lambliasis). ECDC Annual Epidemiological Report for 2019. Stockholm: ECDC. 2022. Available from: https://www.ecdc.europa.eu/en/publications-data/giardiasis-lambliasis-annual-epidemiological-report-2019

[3] Krumrie S Capewell P Smith-Palmer A Mellor D Weir W Alexander CL . A scoping review of risk factors and transmission routes associated with human giardiasis outbreaks in high-income settings. Curr Res Parasitol Vector Borne Dis. 2022;2:100084. 10.1016/j.crpvbd.2022.100084 36589877 PMC9795371

[4] European Centre for Disease Prevention and Control. Surveillance systems overview for 2019. Annual Epidemiological Report for 2019. Stockholm: ECDC; 2022. Available from: https://www.ecdc.europa.eu/en/publications-data/surveillance-systems-overview-2019

[5] Escobedo AA Almirall P Hanevik K Cimerman S Rodríguez-Morales AJ Almanza C Giardiasis: a diagnosis that should be considered regardless of the setting. Epidemiol Infect. 2018;146(10):1216-8. 10.1017/S0950268818001504 29886858 PMC9134294

[6] Espelage W an der Heiden M Stark K Alpers K . Characteristics and risk factors for symptomatic Giardia lamblia infections in Germany. BMC Public Health. 2010;10(1):41. 10.1186/1471-2458-10-41 20105338 PMC2824735

[7] Faensen D Claus H Benzler J Ammon A Pfoch T Breuer T SurvNet@RKI - a multistate electronic reporting system for communicable diseases. Euro Surveill. 2006;11(4):7-8. 10.2807/esm.11.04.00614-en 29208145

[8] Tennekes M . tmap: Thematic Maps in R. J Stat Softw. 2018;84(6):1-39. 10.18637/jss.v084.i06 30450020

[9] Robert Koch Institute (RKI). Infektionsepidemiologisches Jahrbuch meldepflichtiger Krankheiten für 2019. [Infectious disease epidemiological yearbook for 2019] Berlin: RKI; 2019. German. Available from: https://www.rki.de/DE/Content/Infekt/Jahrbuch/Jahrbuch_2019.html

[10] European Centre for Disease Prevention and Control (ECDC). Giardiasis (lambliasis). In: ECDC Annual Epidemiological Report for 2017. Stockholm: ECDC. 2019. Available from: https://www.ecdc.europa.eu/en/publications-data/giardiasis-lambliasis-annual-epidemiological-report-2017

[11] Coffey CM Collier SA Gleason ME Yoder JS Kirk MD Richardson AM Evolving epidemiology of reported giardiasis cases in the United States, 1995-2016. Clin Infect Dis. 2021;72(5):764-70. 10.1093/cid/ciaa128 32047932 PMC9651178

[12] Sagebiel D Weitzel T Stark K Leitmeyer K . Giardiasis in kindergartens: prevalence study in Berlin, Germany, 2006. Parasitol Res. 2009;105(3):681-7. 10.1007/s00436-009-1438-5 19404678

[13] Gebel J Teichert-Barthel U Hornbach-Beckers S Vogt A Kehr B Littmann M Hygiene-Tipps für Kids. [Hygiene tips for kids. Concept and examples of realisation]. Bundesgesundheitsblatt Gesundheitsforschung Gesundheitsschutz. 2008;51(11):1304-13. German. 10.1007/s00103-008-0697-0 19043759

[14] Heudorf U . Hygiene und Infektionsprävention in medizinischen Einrichtungen und in Kindergemeinschaftseinrichtungen - Gesetzliche Grundlagen, Überwachungspraxis und Erfahrungen der Gesundheitsämter. [Hygiene and infection prevention in medical institutions, kindergartens and schools - statutory basis, infection control practice and experiences of the public health services]. Gesundheitswesen. 2015;77(7):481-7. German. 26154256 10.1055/s-0035-1550021

[15] Robert Koch Institut (RKI). Robert Koch-Institut: SurvStat@RKI 2.0. Berlin: RKI. [Accessed: 1 Mar 2022]. Available from: https://survstat.rki.de/Content/Query/Create.aspx

[16] Destatis (Federal Statistical Office of Germany). Pressemitteilung Nr. 451 vom 21. Oktober 2022 [Press release no. 451 from 21 October 2022]. Wiesbaden: Statistisches Bundesamt; 2022. Available from: https://www.destatis.de/DE/Presse/Pressemitteilungen/2022/10/PD22_451_225.html

[17] Yoder JS Gargano JW Wallace RM Beach MJ Centers for Disease Control and Prevention (CDC) . Giardiasis surveillance--United States, 2009-2010. MMWR Surveill Summ. 2012;61(5):13-23. 22951494

[18] Ferguson LC Smith-Palmer A Alexander CL . An update on the incidence of human giardiasis in Scotland, 2011-2018. Parasit Vectors. 2020;13(1):291. 10.1186/s13071-020-04160-9 32513243 PMC7282119

[19] Escobedo AA Almirall P Alfonso M Cimerman S Chacín-Bonilla L . Sexual transmission of giardiasis: a neglected route of spread? Acta Trop. 2014;132:106-11. 10.1016/j.actatropica.2013.12.025 24434784

[20] Fernández-Huerta M Zarzuela F Barberá MJ Arando M Esperalba J Rodríguez V Sexual transmission of intestinal parasites and other enteric pathogens among men who have sex with men presenting gastrointestinal symptoms in an STI unit in Barcelona, Spain: a cross-sectional study. Am J Trop Med Hyg. 2019;101(6):1388-91. 10.4269/ajtmh.19-0312 31549611 PMC6896874

[21] Herbinger KH Alberer M Berens-Riha N Schunk M Bretzel G von Sonnenburg F Spectrum of imported infectious diseases: a comparative prevalence study of 16,817 German travelers and 977 immigrants from the tropics and subtropics. Am J Trop Med Hyg. 2016;94(4):757-66. 10.4269/ajtmh.15-0731 26903611 PMC4824215

[22] Vespestad MK Mehmetoglu M . Gender differences in vacation behavior. Tour Rev Int. 2015;19(3):147-61. 10.3727/154427215X14430967453670

[23] Gallas-Lindemann C Sotiriadou I Plutzer J Karanis P . Prevalence and distribution of Cryptosporidium and Giardia in wastewater and the surface, drinking and ground waters in the Lower Rhine, Germany. Epidemiol Infect. 2013;141(1):9-21. 10.1017/S0950268812002026 23010178 PMC9152036

[24] Naumova EN Jagai JS Matyas B DeMaria A Jr MacNeill IB Griffiths JK . Seasonality in six enterically transmitted diseases and ambient temperature. Epidemiol Infect. 2007;135(2):281-92. 10.1017/S0950268806006698 17291363 PMC2870561

[25] Dietrich J Hammerl JA Johne A Kappenstein O Loeffler C Nöckler K Impact of climate change on foodborne infections and intoxications. J Health Monit. 2023;8(Suppl 3):78-92. 37342431 10.25646/11403 PMC10278375

[26] Lal A Baker MG Hales S French NP . Potential effects of global environmental changes on cryptosporidiosis and giardiasis transmission. Trends Parasitol. 2013;29(2):83-90. 10.1016/j.pt.2012.10.005 23219188

[27] Woschke A Faber M Stark K Holtfreter M Mockenhaupt F Richter J Suitability of current typing procedures to identify epidemiologically linked human Giardia duodenalis isolates. PLoS Negl Trop Dis. 2021;15(3):e0009277. 10.1371/journal.pntd.0009277 33764999 PMC8023459

[28] Ankarklev J Lebbad M Einarsson E Franzén O Ahola H Troell K A novel high-resolution multilocus sequence typing of Giardia intestinalis Assemblage A isolates reveals zoonotic transmission, clonal outbreaks and recombination. Infect Genet Evol. 2018;60:7-16. 10.1016/j.meegid.2018.02.012 29438742

[29] Klotz C Sannella AR Weisz F Chaudhry U Sroka J Tůmová P Extensive testing of a multi-locus sequence typing scheme for Giardia duodenalis assemblage A confirms its good discriminatory power. Parasit Vectors. 2022;15(1):489. 10.1186/s13071-022-05615-x 36572928 PMC9791779

[30] Hommes F Dörre A Behnke SC Stark K Faber M . Travel-related giardiasis: incidence and time trends for various destination countries. J Travel Med. 2023;30(6):taad107. 10.1093/jtm/taad107 37561417 PMC10628773

[31] Ullrich A Schranz M Rexroth U Hamouda O Schaade L Diercke M Impact of the COVID-19 pandemic and associated non-pharmaceutical interventions on other notifiable infectious diseases in Germany: An analysis of national surveillance data during week 1-2016 - week 32-2020. Lancet Reg Health Eur. 2021;6:100103. 10.1016/j.lanepe.2021.100103 34557831 PMC8454829

[32] European Centre for Disease Prevention and Control (ECDC). ECDC Surveillance Atlas. Stockholm: ECDC. [Accessed: 7 Sep 2023]. Available from: https://atlas.ecdc.europa.eu/public/index.aspx

[33] Robert Koch Institute (RKI). Falldefinitionen des Robert Koch-Instituts zur Übermittlung von Erkrankungs- oder Todesfällen und Nachweisen von Krankheitserregern. [The 2019 edition of case definitions for the surveillance of disease, mortality and detection of pathogens.] Berlin: RKI; 2019. German. Available from: https://www.rki.de/DE/Content/Infekt/IfSG/Falldefinition/falldefinition_node.html

